# Summary report MTAA14–NAMLS11

**DOI:** 10.1007/s10967-016-4870-x

**Published:** 2016-05-25

**Authors:** Peter Bode, Antonia Denkova

**Affiliations:** Delft University of Technology, Delft, The Netherlands

The 14th International Conference on Modern Trends in Activation Analysis, MTAA14, and the 11th International Conference on Nuclear Analytical Methods in the Life Science, NAMLS11, were held consecutively from August 23 2015, in Delft, The Netherlands, at the premises of the Delft University of Technology. MTAA14 was chaired by Peter Bode; NAMLS11 was chaired by Antonia Denkova, both from the Delft University of Technology. Professor Marcel de Bruin (same university) was honorary chair of MTAA14.

**Figure:**
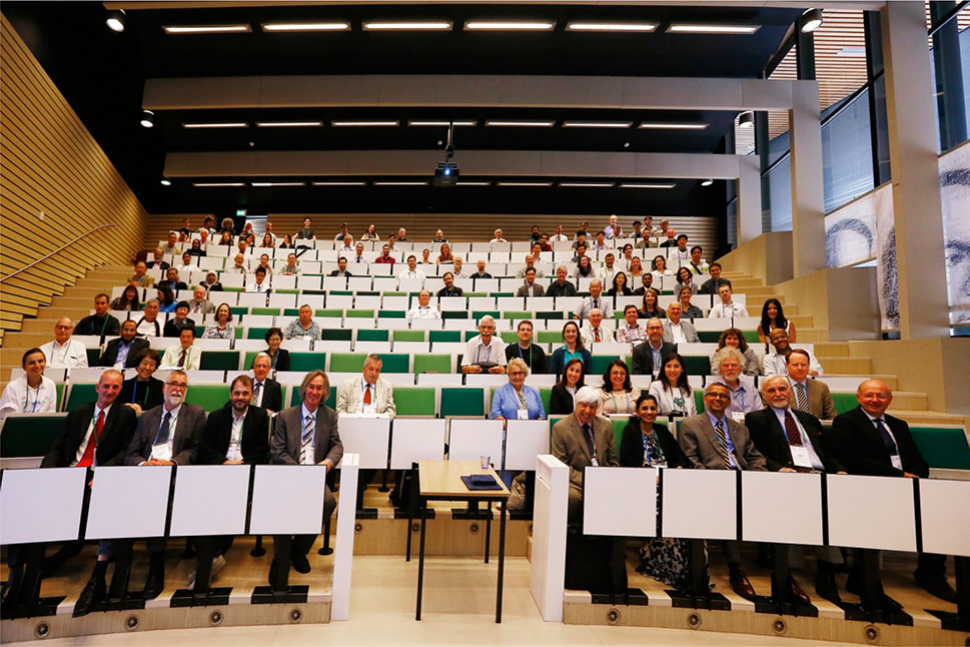
Group picture

The theme of MTAA14 was on Innovation–Relevance–Sustainability; the theme of NAMLS11 was on Innovation–Relevance–Synergy. Each conference had its own scientific committee, assisting the conference chairs in planning the scientific scope, reviewing the abstracts and recommending for oral or poster presentation.

The number of registered participants for both events was 141. In total 245 abstracts were received, 2 abstract were rejected, 30 were withdrawn, and 213 abstracts were accepted from the registered participants. These abstracts were presented in 96 oral presentation (85 ‘regular’ orals, 11 short orals) and as 117 posters.

The conference organization supported participation of 33 persons, including 13 students, by full or partial waiving of their registration fees.

**Figure:**
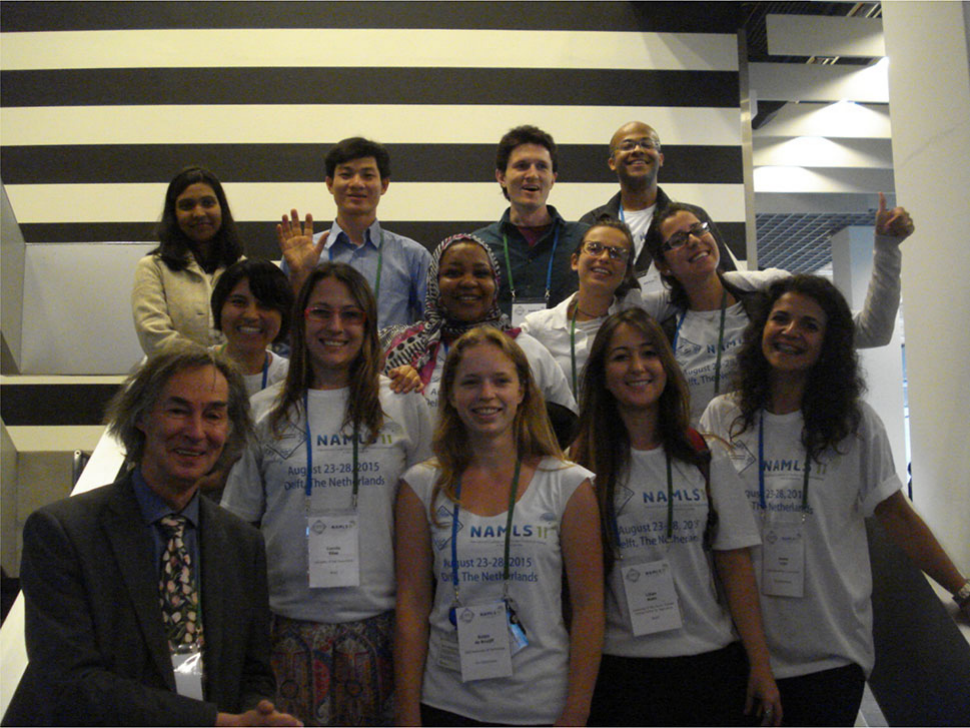
Student crew

In 2016 two Hevesy Medal Awards were facilitated by the Journal of Radioanalytical and Nuclear Chemistry Board of the Hevesy Award. In Delft, Professor Kattesh V. Katti, University of Missouri Cancer Nanotechnology Platform, Columbia, MO, United States of America received his Hevesy Medal in recognition of his innovative contributions in the fields of radiopharmaceuticals and toward the development of nanomedicine products. The other award to Professor Susanta Lahiri was given already in April 2016 at the MARC-X conference. Professor Katti presented in Delft his award lecture on “Theranostic Gold-198 Nanoparticles In Nanomedicine: Implications In Concurrent Molecular Imaging and Tumor Therapy”.

The conferences were preceded on Sunday morning August 23 by two tutorial sessions on (1) “Neutron Activation Analysis: Metrological Principles” by Robert R. Greenberg, National Institutes of Standards and Technology, Gaithersburg, MD, USA; and (2) “Neutron activation analysis: The practice and trouble-shooting” by Peter Bode, Delft University of Technology, The Netherlands.

**Figure:**
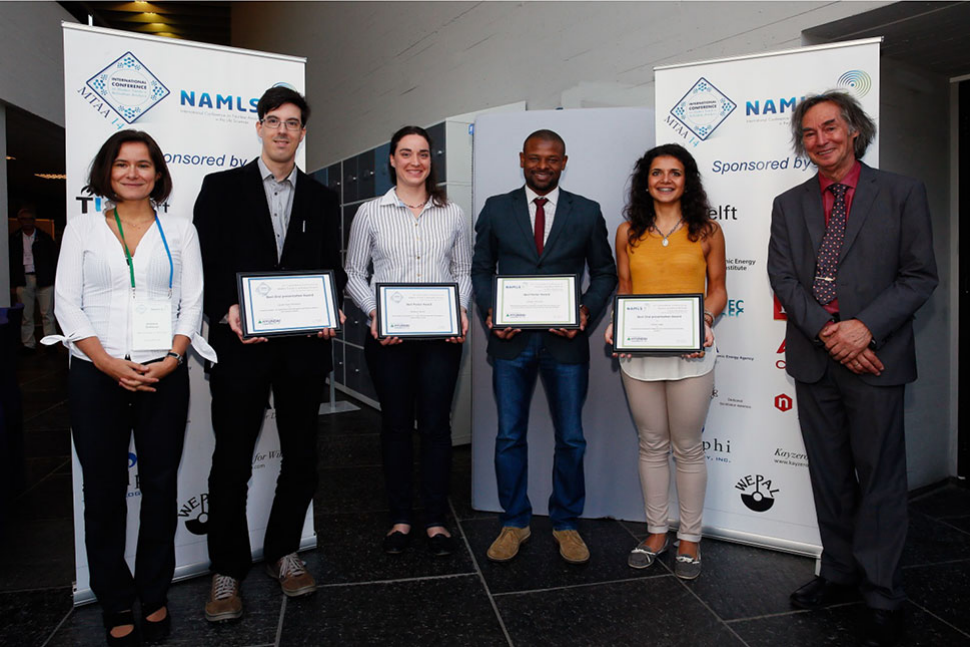
Winners of best oral/poster (*left* to *right*) Antonia Denkova, chair NAMLS11, László Szentmiklósi, Barbara Karches, Johann Antoine, Joana Lage, Peter Bode, chair MTAA14

There were several starting points in the organization of these conferences, some leading to new approaches in the traditional program of the MTAA and NAMLS conference series:

- A conference program aiming not just for listening, but with time to talk to each other. One of the prime objectives of a scientific conference is ‘to confer’. The program was therefore organized around long coffee and lunch breaks, and with long poster sessions, thus providing ample time for discussions.
- Young scientists as session chairs. A new generation has to be stimulated to take the lead. The oral presentations were largely grouped in three simultaneous sessions. Young scientists, including students, were invited to act as session chair to build up experience, with experienced co-chaired acting as a mentor. This initiative was highly appreciated by all involved.
- Invited presentations by outsiders for a broader orientation and reflection on what’s happening in other fields relevant to activation analysis and NAMLS. Six invited presentations were included in the program. These plenary presentations were set at the end of the morning sessions rather than at the beginning, as commonly is done. This new approach allowed for immediate further discussions on the topics presented during the lunch breaks. Scientists were invited from the applied fields, or dealing with instrument development, innovative nuclear facilities and from complementary analytical techniques: (1) “Beyond the determination of accurate, elemental concentrations: complementary analytical approaches in life sciences” by Petra Krystek, University Amsterdam, The Netherlands; (2) “Needs for analytical methods to detect nanomaterials in biological tissues—critical information in risk assessment” by Flemming R. Cassee, National Institute for Public Health and the Environment Bilthoven, The Netherlands; (3) “Trace element analysis: balancing cost, speed and detection limit with the need for reliable, globally accepted measurements” by Michael Sargent, LGC Ltd, Teddington, United Kingdom; (4) “Application of synchrotron techniques for studying the uptake and transformation of nanomaterials in biological systems” by Chunying Chen, National Center for Nanoscience and Technology of China, Beijing, China; (5) “New developments in scintillation detectors seen from an industrial perspective: current trends and expectations for the future” by Paul Schotanus, Scionix Nederland, The Netherlands; and (6) “Prompt gamma-ray analysis using pulsed neutrons at J-PARC” by Yosuke Toh, Japan Atomic Energy Agency, Ibaraki, Japan.
- Short oral sessions. Several participants received a timeslot of 5 min to present their work in the plenary meeting room. This approach was well received by all; firstly because a large audience has the opportunity to listen to all presentations and secondly, because a conference program can thus cover many oral presentations without the need for more parallel sessions, thus opening more time for other scientific activities. All presenters were able to explain in those 5 min the objectives, approaches, results and conclusions of their research and this approach was very well received.

The poster sessions were preceded by 1 min presentations in the plenary meeting room; these presentations served as teasers to come and see the posters.

In addition, the program included an Editor’s forum. Zsolt Revay (J. Radioanal.Nucl.Chem.), Syed M. Qaim (Radiochim. Acta), Flemming Cassee (Particle Fiber Technology), Kattesh V. Katti (J. Green Nanotechnology), Bert Wolterbeek (Environmental Pollution) and Adrie Bos (Radiation Measurements) presented the policies of the various journals on reviewing and accepting manuscripts for publication, and answered questions from the floor.

In a hands-on software workshop under supervision of Menno Blaauw participants had an opportunity to discuss with experts and software manufactures problems from the daily practice, learn about new opportunities and compare the various packages.

Dick Lindstrom and Marcel de Bruin were moderators in a brainstorming session on the current status of activation analysis, and ways to go forward. Both events were well visited.

The conferences were sponsored by the following organizations:

Delft University of Technology, Reactor Institute Delft, International Atomic Energy Agency, Korean Atomic Energy Research Institute-Hyundai Engineering & Construction Consortium, Hyundai Engineering Co. Ltd., Dr. Westmeier GmbH, Wageningen Evaluating Programmes for Analytical Laboratories, BRIGHTSPEC, CANBERRA, SCIONIX-CAEN, Kayzero for Windows, Adelphi Technology Inc.

The sponsorship facilitated not only the organization of the conferences but also the awards for best oral and best poster presentation in the MTAA14 and NAMLS11 sessions. Following recommendations by session chairs and co-chairs, the following contributions were awarded: (1) Best poster NAMLS11: Mr. Johann Antoine, International Centre for Environmental and Nuclear Sciences, Kingston, Jamaica, for “Sourcing the provenance of Jamaican Cannabis from four locations by elemental profiling using Instrumental Neutron Activation Analysis”; (2) Best poster MTAA14: Ms. Barbara Karches, Universität Mainz, Germany, for “Determination of phosphorus in n-type silicon by INAA with a beta–gamma anticoincidence system”; (3) Best oral presentation NAMLS11: Ms. Joana Lage, Delft University of Technology, The Netherlands and Instituto Superior Técnico, Lisbon, Portugal, for “Assessment of a Steelwork Impact by Instrumental Neutron Activation Analysis” and (4) Best oral presentation MTAA14: Mr. László Szentmiklósi, Centre for Energy Research, Hungarian Academy of Sciences, Budapest, Hungary, for “In-beam catalysis—an application of prompt-gamma activation analysis in the catalysis research”.

A technical tour the Reactor Institute Delft with visit to the research reactor and NAA facilities was included in the program,

The conference organization facilitated meetings of the NAMLS International Committee, the k-0 International Scientific Committee and the International Committee on Activation Analysis.

The social program of the conference included a welcoming reception by the Lord Mayor Bas Verkerk of the city of Delft at the Delft Town hall, who also gave a well-received inspiring speech.; an excursion to Madurodam in The Hague, followed by the conference dinner; the live preparation of, and tasting of ‘stroopwafels’—a world-famous Dutch delight-, and a farewell reception.

The logo’s and house style for MTAA14 and NAMLS11 were developed by Linda Van der Meer, LIN! Grafisch Ontwerp.

MTAA14 and NAMLS11 could be organized by support of the municipality of Delft, the Delft University of Technology—especially the Dean, Prof. Dr. Ir. Rob Fastenau and Facility Management of the Faculty of Electrical Engineering, Mathematics and Computer Science for their hospitality to facilitate the conferences within their premises; by the Reactor Institute Delft (especially Jose Buurman, the conference secretary) and, not in the least, by the enthusiastic, creative and open minded people of Delft Event Solutions: Dina Konings, Marjo van Koppen and Natascha Voskuijl and the student-participant crew under supervision of Robin de Kruijff during the conferences itself.

We thank all for supporting us in organizing the conferences, and for participation and coming to Delft.

